# Comparison of VTE risk scores in guidelines for VTE diagnosis in nonsurgical hospitalized patients with suspected VTE

**DOI:** 10.1186/s12959-023-00450-1

**Published:** 2023-01-19

**Authors:** Wei Xiong, Yunfeng Zhao, Yi Cheng, He Du, Jinyuan Sun, Yanmin Wang, Mei Xu, Xuejun Guo

**Affiliations:** 1 Department of Pulmonary and Critical Care Medicine, Xinhua Hospital, Shanghai Jiaotong University School of Medicine, No. 1665, Kongjiang Road, Yangpu District, Shanghai, 200092 China; 2 Department of Pulmonary and Critical Care Medicine, Pudong New District, Punan Hospital, Shanghai, China; 3 Department of Medical Oncology, Shanghai Pulmonary Hospital, Tongji University School of Medicine, Shanghai, China; 4 Department of General Practice, North Bund Community Health Service Center, Hongkou District, Shanghai, China

**Keywords:** Venous thromboembolism, Nonsurgical hospitalized patients, Guidelines, Risk score, Diagnosis, Predictive power

## Abstract

**Background:**

The assessment of VTE likelihood with VTE risk scores is essential prior to imaging examinations during VTE diagnostic procedure. Little is known with respect to the disparity of predictive power for VTE diagnosis among VTE risk scores in guidelines for nonsurgical hospitalized patients with clinically suspected VTE.

**Methods:**

A retrospective study was performed to compare the predictive power for VTE diagnosis among the Wells, Geneva, YEARS, PERC, Padua, and IMPROVE scores in the leading authoritative guidelines in nonsurgical hospitalized patients with suspected VTE.

**Results:**

Among 3168 nonsurgical hospitalized patients with suspected VTE, VTE was finally excluded in 2733(86.3%) ones, whereas confirmed in 435(13.7%) ones. The sensitivity and specificity resulted from the Wells, Geneva, YEARS, PERC, Padua, and IMPROVE scores were (90.3%, 49.8%), (88.7%, 53.6%), (73.8%, 50.2%), (97.7%,16.9%), (80.9%, 44.0%), and (78.2%, 47.0%), respectively. The YI were 0.401, 0.423, 0.240, 0.146, 0.249, and 0.252 for the Wells, Geneva, YEARS, PERC, Padua, and IMPROVE scores, respectively. The C-index were 0.694(0.626–0.762), 0.697(0.623–0.772), 0.602(0.535–0.669), 0.569(0.486–0.652), 0.607(0.533–0.681), and 0.609(0.538–0.680) for the Wells, Geneva, YEARS, PERC, Padua, and IMPROVE scores, respectively. Consistency was significant in the pairwise comparison of Wells vs Geneva(Kappa 0.753, *P* = 0.565), YEARS vs Padua(Kappa 0.816, *P* = 0.565), YEARS vs IMPROVE(Kappa 0.771, *P* = 0.645), and Padua vs IMPROVE(Kappa 0.789, *P* = 0.812), whereas it did not present in the other pairs. The YI was improved to 0.304, 0.272, and 0.264 for the PERC(AUC 0.631[0.547–0.714], *P* = 0.006), Padua(AUC 0.613[0.527–0.700], *P* = 0.017), and IMPROVE(AUC 0.614[0.530–0.698], *P* = 0.016), with a revised cutoff of 5 or less, 6 or more, and 4 or more denoting the VTE-likely, respectively.

**Conclusions:**

For nonsurgical hospitalized patients with suspected VTE, the Geneva and Wells scores perform best, the PERC scores performs worst despite its significantly high sensitivity, whereas the others perform intermediately, albeit the absolute predictive power of all isolated scores are mediocre. The predictive power of the PERC, Padua, and IMPROVE scores are improved with revised cutoffs.

## Introduction

Venous thromboembolism(VTE) consistsof pulmonary embolism(PE), deep venous thrombosis (DVT), superficial vein thrombosis (SVT), and splanchnic vein thrombosis (SPVT) [1]. In general, VTE mainly refers to PE and DVT [2]. VTE is the third most frequent global acute cardiovascular syndrome behind myocardial infarction and stroke [3]. Annual incidence rate of PE ranges from 39–115 per 100 000 population, whereas incidence rate of DVT ranges from 53–162 per 100 000 population [4, 5]. PE causes almost 300 000 deaths per year in the US, ranking high among all causes of cardiovascular mortality [4].

Strong risk factors for VTE occurrence mainly comprise but not limited to: active cancer, previous VTE, antiphospholipid syndrome, recent hospitalization for acute illness especially myocardial infarction, heart failure or atrial fibrillation/flutter, recent major trauma or fracture or surgery especially hip or knee replacement, prolonged immobility > 3 days, and heparin-induced thrombocytopenia. Clinical assessment for predisposing risk factors and presentation of symptoms of VTE allows the stratification of patients with suspected VTE into distinct categories which correspond to an actual prevalence of confirmed VTE, and is necessary to estimate patients’ risk of VTE before any further investigations. Pretest probability(PTP) assessment of VTE is the first and key step throughout the the whole diagnostic algorithms for VTE, since the post-test probability of VTE or the interpretation of results of imaging testings depends not only on the results itself but also on the pretest probability of VTE. Risk assessment of VTE can be performed either by using empirical clinical gestalt or by using standardized models [2, 6]. Notwithstanding the value of empirical clinical gestalt has been confirmed in several large studies [7, 8], standardized VTE risk assessment using clinical models or scores or rules is preferred over gestalt, since gestalt lacks standardization or the possibility of imparting standard operating procedure [2, 6, 9, 10].

Accordingly, a series of VTE(including PE) risk scores which were represented by the Wells score [11, 12] and the revised Geneva score [13, 14] have emerged one after another. An independent VTE risk score usually consists of VTE risk factors, weighing points of risk factors, and defined cutoffs for risk classifications. By far, the VTE risk scores which have been approved by leading authoritative guidelines such as the European Society of Cardiology (ESC)/European Respiratory Society (ERS), the American College of Chest Physicians(ACCP) and the American Society of Hematology(ASH) for patients with suspected VTE mainly include the Wells [2, 9, 10, 15, 16], the revised Geneva [2, 9, 10, 15], the YEARS [2], the PERC [2], the Padua [17, 18], the IMPROVE [18], the Caprini [19], and the Rogers [19]. Since the latter two are completely targeted for surgical patients, their VTE risk assessment value in nonsurgical patient population are limited. In addition, notwithstanding there is a Geneva VTE risk assessment model (RAM) [20, 21], it has not been endorsed by primary authoritative guidelines by far.

To our best knowledge, no study ever compared the predictive power for VTE diagnosis among all these VTE risk scores approved by the leading authoritative guidelines for nonsurgical hospitalized patients with suspected VTE to date. However, clinicians may yield confusion of how to choose them in daily clinical practice, facing a variety of VTE risk scores. Accordingly, the present study was carried out to address this issue.

## Methods

### Study design

A retrospective study was performed to compare the predictive power for VTE diagnosis among six VTE risk scores including the Wells, Geneva, YEARS, PERC, Padua, and IMPROVE RAMs which are approved by the leading authoritative guidelines for nonsurgical hospitalized patients with suspected VTE. Nonsurgical hospitalized patients were reviewed if they had undergone diagnostic imaging investigation of VTE that included computed tomography pulmonary angiography (CTPA), compression ultrasonography (CUS) of lower extremities, and/or planar ventilation/perfusion (V/Q) scan due to the suspicion of VTE which were triggered by typical symptoms or signs of PE and/or DVT, and/or a D-dimer level was 500 ng/mL or more. Clinical suspicion of VTE was yielded by patients’ attending physicians at the admission of hospitalizations. VTE was defined as PE and DVT. Nonsurgical patients were defined as patients who were not in a perioperative period. All eligible patients were classified into VTE and non-VTE groups according to their results of VTE imaging examinations. During the present study, the PTP of VTE in each patient was reassessed with the Wells, Geneva, YEARS, PERC, Padua, and IMPROVE scores, respectively. The results of VTE likelihood assessment by each score in each patient was defined as either VTE-unlikely or VTE-likely, respectively. Then such VTE unlikeliness or likeliness resulted from all scores was contrasted to the actual absence or presence of VTE for all patients, thereby comparing their predictive power for VTE diagnosis. The pairwise comparison of diagnostic consistency and dominance were conducted between very two RAMs. The predictive power for VTE diagnosis was reanalyzed without using the originally-defined cutoffs of all scores, to explore whether or not their performance would be improved with a revised cutoff, thereby validating the appropriateness of their original cutoffs. The parameters at the time of hospital admission were harvested as the variables involved in RAMs in the present study.

**Fig. 1:**
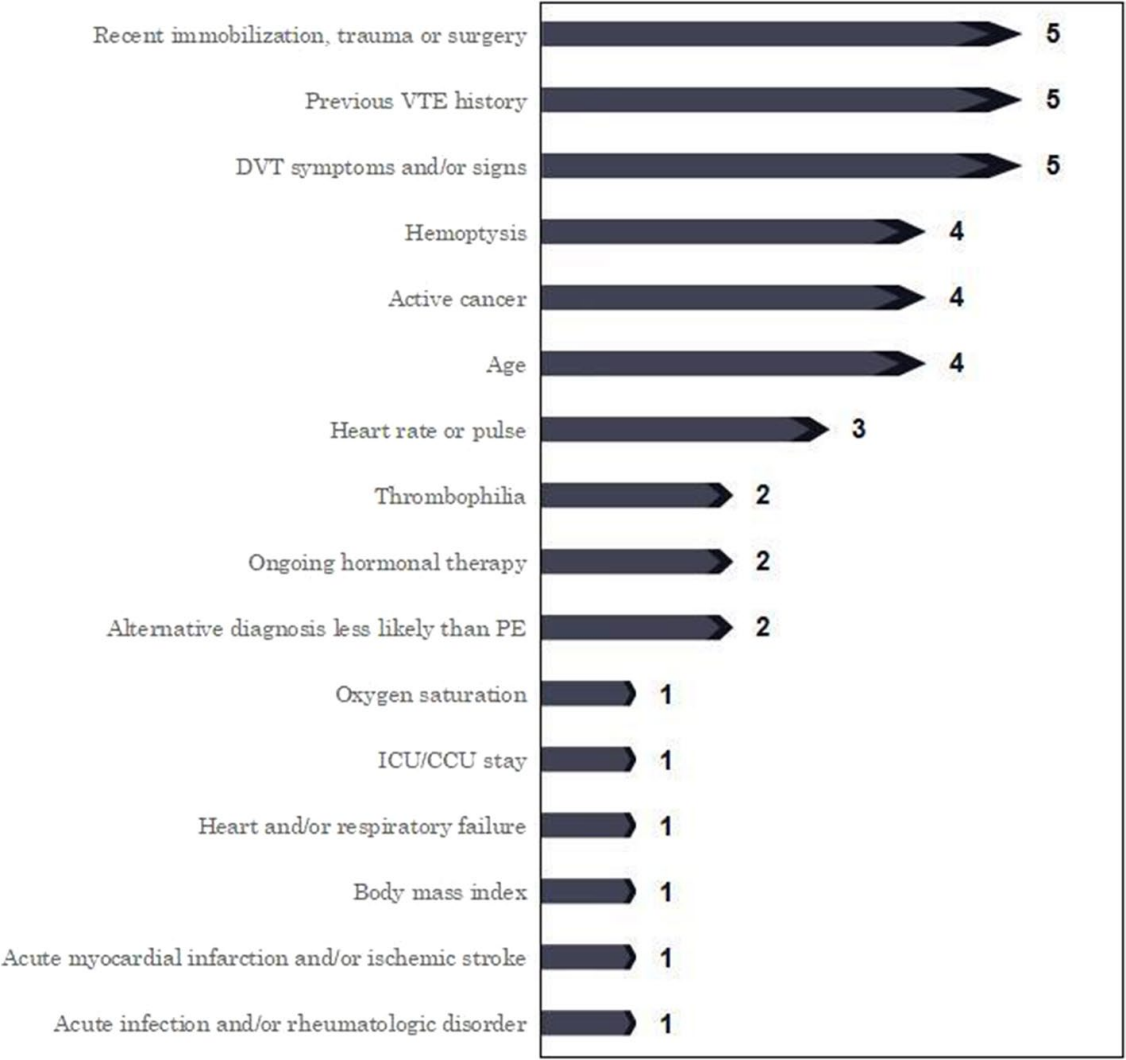
Presence Frequency of VTE Risk Elements of all Scores in Descending Order. Note: VTE: Venous thromboembolism, PE: Pulmonary embolism, DVT: Deep venous thrombosis, ICU/CCU: Intensive care unit/coronary care unit

With respect to the Wells score, the simplified version was adopted in the current study due to its increased adoption into clinical practice than the original one [22], albeit the term of “Wells” is still used for the rest of this article. It consists of previous PE or DVT(1 point), heart rate > 100 beats per minute (bpm)(1 point), surgery or immobilization within the past 4 weeks(1 point), hemoptysis(1 point), active cancer(1 point), clinical signs of DVT(1 point), and alternative diagnosis less likely than PE(1 point). A total score of 1 or less denotes VTE-unlikely, whereas 2 or more denotes VTE-likely [2]. Likewise, the simplified revised version was employed for the Geneva score for the same reason [23], albeit the term of “Geneva” is still used for the rest of this article. It contains previous PE or DVT(1 point), heart rate75-94 bpm(1 point), heart rate ≥ 95 bpm(2 points), surgery or fracture within the past month(1 point), hemoptysis(1 point), active cancer(1 point), unilateral lower-limb pain(1 point), pain on lower-limb deep venous palpation and unilateral oedema(1 point), age > 65 years(1 point). A total score of 2 or less denotes VTE-unlikely, whereas 3 or more denotes VTE-likely [2]. The YEARS score consists of clinical signs of DVT(1 point), hemoptysis(1 point), and PE is the most likely diagnosis(1 point). A total score of 0 denotes VTE-unlikely, whereas 1 or more denotes VTE-likely [2, 6, 24]. The PERC rule comprises age < 50 years(1 point), pulse < 100 bpm(1 point), oxygen saturation(SaO_2_) > 94%(1 point), no unilateral leg swelling(1 point), no hemoptysis(1 point), no recent trauma or surgery(1 point), no history of VTE(1 point) and no oral hormone use(1 point). A total score of 8 denotes VTE-unlikely, whereas 7 or less denotes VTE-likely [2, 25]. The Padua score contains reduced mobility(3 points), active cancer(3 points), previous VTE excluding superficial thrombophlebitis(3 points), known thrombophilic condition(3 points), recent trauma and/or surgery within the past month(2 points), age > 70 years(1 point), heart and/or respiratory failure(1 point), acute myocardial infarction or ischemic stroke(1 point), ongoing hormonal treatment(1 point), body mass index > 30(1 point), acute infection and/or rheumatologic disorder(1 point). A total score of 3 or less denotes VTE-unlikely, whereas 4 or more denotes VTE-likely [17, 18]. The IMPROVE score consists of previous VTE(3 points), known thrombophilia(2 points), lower limb paralysis(2 points), active cancer(2 points), immobilization ≥ 7 days(1 point), intensive care unit(ICU)/coronary care unit(CCU) stay(1 point), age > 60 years(1 point). A total score of 1 or less denotes VTE-unlikely, whereas 2 or more denotes VTE-likely [18]. The summary of characteristics of all six scores are presented in Table 1. The presence frequency of VTE risk elements in all six scores in descending order are demonstrated in Fig. 1.

**Table 1:** Characteristics of all six VTE Scores

Elements	Wells(7 elements)	Geneva(7 elements)	YEARS(3 elements)	PERC(8 elements)	Padua(10 elements)	IMPROVE(7 elements)
Age(4 times)	-	+	-	+	+	+
Active cancer(4 times)	+	+	-	-	+	+
Alternative diagnosis less likely than PE(2 times)	+	-	+	-	-	-
Acute infection and/or rheumatologic disorder(1 time)	-	-	-	-	+	-
Acute myocardial infarction and/or ischemic stroke(1 time)	-	-	-	-	+	-
Body mass index(1time)	-	-	-	-	+	-
DVT symptoms and/or signs(5 times)	+	+	+	+	-	+
Hemoptysis(4 times)	+	+	+	+	-	-
Heart and/or respiratory failure(1 time)	-	-	-	-	+	-
Heart rate or pulse(3 times)	+	+	-	+	-	-
ICU/CCU stay(1 time)	-	-	-	-	-	+
Ongoing hormonal therapy(2 times)	-	-	-	+	+	-
Oxygen saturation(1 time)	-	-	-	+	-	-
Previous VTE history(5 times)	+	+	-	+	+	+
Recent immobilization, trauma or surgery(5 times)	+	+	-	+	+	+
Thrombophilia(2 times)	-	-	-	-	+	+
Presence frequency sum of VTE risk elements(times)	28	30	11	29	26	26
Presence frequency per VTE risk element(times)	4.00	4.29	3.67	3.63	2.60	3.71
Total points	7	9	3	8	20	12
Cutoff points	VTE unlikely 0–1 VTE likely ≥ 2	VTE unlikely 0–2 VTE likely ≥ 3	VTE unlikely 0 VTE likely ≥ 1	VTE unlikely 8 VTE likely ≤ 7	VTE unlikely 0–3 VTE likely ≥ 4	VTE unlikely 0–1 VTE likely ≥ 2
Ratio of VTE-likely cutoff points to total points	0.29	0.33	0.33	0.88	0.20	0.17

*VTE* Venous thromboembolism, *PE* Pulmonary embolism, *DVT* Deep venous thrombosis, *ICU/CCU* Intensive care unit/coronary care unit

“ + ” denotes the presence of the VTE risk elements, “-” denotes the absence of the VTE risk elements

The present study was conducted by the investigators of Shanghai Xinhua Hospital, Shanghai Pulmonary Hospital, and Shanghai Punan Hospital. Relevant data were retrieved from the electronic medical record systems of each participating hospital. All authors vouched for the completeness and accuracy of the data. No one who is not an author contributed to the manuscript writing. The study protocol was approved by the institutional review board of each participating hospital.

### Study population

Eligible patients from participating hospitals were incorporated into the present study as per the inclusion and exclusion criteria. The inclusion criteria consisted of the following:1) All eligible patients were 18 years old or older; 2) All eligible patients underwent diagnostic imaging investigation of VTE that included CTPA, CUS of lower extremities, and/or planar V/Q scan to confirm the absence or presence of VTE during the hospitalization;3) All eligible patients were nonsurgical hospitalized ones. The exclusion criteria consisted of the following:1) Patients were excluded if they had a known previous history of chronic thromboembolic disease (CTED) [2] or were diagnosed with CTED during the hospitalization; 2) Patients were excluded if they were finally diagnosed with non-thrombotic venous embolism primarily including tumor embolism, amniotic fluid embolism, fat embolism, septic embolism, and angiosarcoma during the hospitalization.

### Statistical analyses

Comparison of measurement data between groups was conducted by using T-test. Comparison of rates was conducted by Chi-square test. Number of patients with true positive (TP), false positive (FP), false negative (FN), and true negative (TN) resulted from each score, sensitivity, specificity, positive predictive value (PPV), negative predictive value (NPV), false positive rate (FPR)(misdiagnosis rate), false negative rate (FNR) (omission diagnostic rate), positive likelihood ratio (PLR), negative likelihood ratio (NLR), diagnostic odds ratio (DOR), number needed to diagnosis(NND), success rate (SR)(crude agreement), failure rate(FR), adjusted agreement (AA), Youden index (YI), and Harrell's concordance-index(C-index) were compared among the Wells, Geneva, YEARS, PERC, Padua, and IMPROVE scores. Pairwise comparison of diagnostic consistency and dominance tests between every two scores were performed by using Cohen's Kappa coefficient analysis and McNemar’s test, respectively. Logistic regression analysis was applied to explore the correlation between VTE occurrence and VTE risk elements in scores. Receiver operator characteristic (ROC) curve analyse was performed to reveal and compare the predictive power for VTE diagnosis among all VTE scores without using the originally-defined cutoffs. Statistical analyses were performed by using SPSS 26. A *P*-value being less than 0.05 denoted statistical significance.

## Results

### Demographics and clinical characteristics of patients

A total of 3317 nonsurgical hospitalized patients with a confirmed absence or presence of VTE from Jan, 2012 through Mar, 2022 from the participating hospitals were incorporated into the present study as per the inclusion criteria. As per the exclusion criteria, 116 patients with CTED, and 33 ones with nonthrombotic venous embolism were excluded. Eventually, a total of 3168 patients entered into the final analyses of the present study.

The median age of all patients was 69.9 years old. The number of female and male patients were 1501 and 1667, respectively. For all 3168 patients, VTE was definitely ruled out in 2733(86.3%) ones, whereas was confirmed in 435(13.7%) ones. Among 435 patients with established VTE, 144 one had isolated PE, 118 ones had isolated DVT, whereas 173 ones had concurrent PE and DVT. Among 317 patients with PE, 18 patients had hemodynamic instability. Six patients died of fatal PE. The demographics and clinical characteristics of all patients were summarized in Table 2.

**Table 2:** Demographics and Characteristics of all Patients

Variables	Non-VTE(*n* = 2733)	VTE(*n* = 435)	*P* value
Age-years	62.5 ± 18.6	77.3 ± 15.7	0.015
Sex(female/male)-no.(%)	1308(47.9)/1425(52.1)	193(44.4)/242(55.6)	0.848
Body mass index-kg/m^2^	22.9 ± 7.2	25.3 ± 10.3	0.125
VTE types			
PE-no.(%)		144(33.1)	
DVT-no.(%)		118(27.1)	
PE&DVT-no.(%)		173(39.8)	
Active cancer(Y/N)-no.(%)	202(7.4)/2531(92.6)	148(34.0)/287(66.0)	< 0.001
Alternative diagnosis less likely than PE(Y/N)-no.(%)	1060(38.8)/1673(61.2)	238(54.7)/197(45.3)	0.005
Acute infection and/or rheumatologic disorder(Y/N)-no.(%)	1203(44.0)/1530(56.0)	254(58.4)/181(41.6)	0.037
Acute myocardial infarction and/or ischemic stroke(Y/N)-no.(%)	301(11.0)/2432(89.0)	78(17.9)/357(82.1)	0.010
D-dimer-ng/mL	878.4 ± 358.9	2468.7 ± 1389.6	< 0.001
DVT symptoms and/or signs(Y/N)-no.(%)	352(12.9)/2381(87.1)	274(63.0)/161(37.0)	< 0.001
Hemoptysis(Y/N)-no.(%)	262(9.6)/2471(90.4)	85(19.5)/350(80.5)	< 0.001
Heart and/or respiratory failure(Y/N)-no.(%)	388(14.2)/2345(85.8)	102(23.4)/333(76.6)	0.001
Heart rate or pulse-bpm	78.9 ± 21.6	105.7 ± 33.4	0.002
ICU/CCU stay(Y/N)-no.(%)	366(13.4)/2367(86.6)	130(29.9)/305(70.1)	< 0.001
Ongoing hormonal therapy(Y/N)-no.(%)	246(9.0)/2487(91.0)	50(11.5)/385(88.5)	0.793
Oxygen saturation-%	96.6 ± 4.5	93.7 ± 7.6	0.912
Previous VTE history(Y/N)-no.(%)	215(7.9)/2518(92.1)	98(22.5)/337(77.5)	< 0.001
Recent immobilization, trauma or surgery(Y/N)-no.(%)	382(14.0)/2351(86.0)	222(51.0)/213(49.0)	< 0.001
Thrombophilia(Y/N)-no.(%)	37(1.4)/2696(98.6)	22(5.1)/413(94.9)	< 0.001

*VTE* Venous thromboembolism, *no.* number, *kg/m*^*2*^ Kilogram/meter^2^, *PE* Pulmonary embolism, *DVT* Deep venous thrombosis, *Y* Yes, *N* No, *ng/mL* Nanogram/milliliter, *bpm* Beats per minute, *ICU/CCU* Intensive care unit/coronary care unit

### Correlation between VTE risk elements in scores and VTE occurrence

An univariate and the subsequent multivariate Logistic regression analyses demonstrated that, most VTE risk elements in all six scores were correlated with VTE occurrence except for the elements of acute infection and/or rheumatologic disorder, body mass index, ongoing hormonal therapy, and oxygen saturation, in the present study population. Correlation between VTE risk elements in scores and VTE occurrence are presented in Table 3.

**Table 3:** Correlation Between VTE Risk Elements in Scores and VTE Occurrence

Variables	Odds ratio (univariate)	*P* value	Odds ratio (multivariate)	*P* value
Age(years)- > 65 vs ≤ 65	1.563(1.248–1.878)	0.023	1.649(1.287–2.011)	0.016
Active cancer-yes vs no	4.379(3.124–5.634)	< 0.001	5.726(4.171–7.281)	< 0.001
Alternative diagnosis less likely than PE-yes vs no	1.732(1.215–2.249)	0.005	2.130(1.474–2.786)	0.001
Acute infection and/or rheumatologic disorder-yes vs no	1.771(1.305–2.237)	0.003	1.446(1.126–1.766)	0.097
Acute myocardial infarction and/or ischemic stroke-yes vs no	1.615(1.026–2.204)	0.002	2.175(1.547–2.803)	0.001
Body mass index(kg/m^2^)- > 30 vs ≤ 30	1.739(1.442–2.236)	0.019	1.378(1.024–1.732)	0.256
DVT symptoms and/or signs-yes vs no	7.174(5.258–9.090)	< 0.001	7.339(5.501–9.177)	< 0.001
Hemoptysis-yes vs no	2.373(1.762–2.984)	0.001	2.580(2.037–3.123)	0.001
Heart and/or respiratory failure-yes vs no	2.249(1.883–2.615)	0.001	2.734(2.251–3.217)	0.001
Heart rate or pulse(bpm)- > 100 vs ≤ 100	2.645(2.173–3.117)	0.001	2.283(1.735–2.831)	0.001
ICU/CCU stay-yes vs no	2.361(1.942–2.780)	0.001	2.588(2.174–3.002)	0.001
Ongoing hormonal therapy-yes vs no	1.633(1.248–2.018)	0.025	1.352(1.171–1.533)	0.119
Oxygen saturation(%)- ≤ 94 vs > 94	1.264(1.026–1.502)	0.585		
Previous VTE history-yes vs no	2.828(2.492–3.164)	< 0.001	3.764(3.150–4.378)	< 0.001
Recent immobilization, trauma or surgery-yes vs no	4.653(3.884–5.422)	< 0.001	5.259(4.743–5.775)	< 0.001
Thrombophilia-yes vs no	2.864(2.125–3.603)	< 0.001	3.377(2.756–3.998)	< 0.001

*VTE* Venous thromboembolism, *PE* Pulmonary embolism, *kg/m*^*2*^ Kilogram/meter^2^, *DVT* Deep venous thrombosis, *bpm* Beats per minute, *ICU/CCU* Intensive care unit/coronary care unit

### Comparison of predictive power for VTE diagnosis among all scores

The unlikeliness or likeliness of VTE reassessed by all scores were compared to the actual VTE absence or presence, respectively. The VTE diagnostic prevalence were 55.7%, 52.2%, 53.1%, 85.1%, 59.4%, and 56.4%, whereas the VTE exclusion prevalence were 44.3%, 47.8%, 46.9%, 14.9%, 40.6%, and 43.6% for the Wells, Geneva, YEARS, PERC, Padua, and IMPROVE, respectively. The odds ratio of VTE occurrence(VTE-likely vs VTE-unlikely) were 7.435(6.124–8.746), 7.314(6.003–8.625), 2.486(1.557–3.415), 7.526(5.385–9.667), 2.887(1.444–4.330), and 2.757(1.649–3.865) for the Wells, revised Geneva, YEARS, PERC, Padua, and IMPROVE, respectively. Based on the number of TP, FP, FN, TN resulted from each score, the actual prevalence of VTE in the stratification of VTE-likely and VTE-unlikely yielded by each score were (22.3%, 3.0%), (23.4%, 3.2%), (19.1%, 7.7%), (15.8%, 2.1%), (18.7%, 6.5%), and (19.0%, 6.9%) for the Wells, Geneva, YEARS, PERC, Padua, and IMPROVE scores, respectively.

The diagnostic sensitivity and specificity were (90.3%, 49.8%), (88.7%, 53.6%), (73.8%, 50.2%), (97.7%,16.9%), (80.9%, 44.0%), and (78.2%, 47.0%) for the Wells, Geneva, YEARS, PERC, Padua, and IMPROVE scores, respectively. The Geneva score had the highest PPV(23.4%), whereas the PERC score had the highest NPV(97.9%). The Geneva score had the lowest FPR(46.4%), whereas the PERC score had the lowest FNR(2.30%). The Geneva score had the highest PLR(1.912), whereas the PERC score had the lowest NLR(0.136). The PERC score had the highest DOR(208.4). The Geneva score had the least NND(2.364). The Geneva score had the highest SR(58.5%), AA(65.6%) and the lowest FR(41.5%). The Geneva score also had the maximum difference(17.0%) between the SR and FR. The YI were 0.401, 0.423, 0.240, 0.146, 0.249, and 0.252 for the Wells, Geneva, YEARS, PERC, Padua, and IMPROVE, respectively. The C-index were 0.694(0.626–0.762), 0.697(0.623–0.772), 0.602(0.535–0.669), 0.569(0.486–0.652), 0.607(0.533–0.681), and 0.609(0.538–0.680) for the Wells, Geneva, YEARS, PERC, Padua, and IMPROVE, respectively. The TP, FP, FN, TN, sensitivity, specificity, PPV, NPV, FPR, FNR, PLR, NLR, DOR, NND, SR, FR, AA, YI, and C-index of all scores are demonstrated in Table 4.

**Table 4:** Comparison of Predictive Power for VTE Diagnosis Among all Scores

Variables	Wells	Geneva	YEARS	PERC	Padua	IMPROVE
Score-points	2.69 ± 2.35	3.58 ± 2.92	1.06 ± 1.16	2.97 ± 2.70	8.04 ± 6.20	4.84 ± 3.81
TP-no	393	386	321	425	352	340
FP-no	1372	1267	1360	2270	1531	1448
FN-no	42	49	114	10	83	95
TN-no	1361	1466	1373	463	1202	1285
DP-%	55.7	52.2	53.1	85.1	59.4	56.4
EP-%	44.3	47.8	46.9	14.9	40.6	43.6
Sensitivity -%	90.3	88.7	73.8	97.7	80.9	78.2
Specificity -%	49.8	53.6	50.2	16.9	44.0	47.0
PPV -%	22.3	23.4	19.1	15.8	18.7	19.0
NPV -%	97.0	96.8	92.3	97.9	93.5	93.1
FPR -%	50.2	46.4	49.8	83.1	56.0	53.0
FNR -%	9.70	11.3	26.2	2.30	19.1	21.8
PLR	1.799	1.912	1.482	1.176	1.445	1.475
NLR	0.195	0.211	0.522	0.136	0.434	0.464
DOR	9.433	6.808	2.789	208.4	5.402	4.033
NND-no	2.494	2.364	4.167	6.849	4.016	3.968
SR-%	55.4	58.5	53.5	28.0	49.1	51.3
FR-%	44.6	41.5	46.5	72.0	50.9	48.7
AA -%	64.9	65.6	58.9	57.1	59.2	59.3
YI	0.401	0.423	0.240	0.146	0.249	0.252
C-index	0.694(0.626–0.762)	0.697(0.623–0.772)	0.602(0.535–0.669)	0.569(0.486–0.652)	0.607(0.533–0.681)	0.609(0.538–0.680)

*VTE* Venous thromboembolism, *no.* number, *TP* True Positive, *FP* False Positive, *FN* False Negative, *TN* True Negative, *DP* Diagnostic Prevalence, *EP* Exclusion Prevalence, *PPV* Positive Predictive Value, *NPV* Negative Predictive Value, *FPR* False Positive Rate, *FNR* False Negative Rate, *PLR* Positive Likelihood Ratio, *NLR* Negative Likelihood Ratio, *DOR* Diagnostic Odds Ratio, *NND* Number Needed to Diagnosis, *SR* Success Rate, *FR* Failure Rate, *AA* Adjusted Agreement, *YI* Youden Index, *C-index* Concordance-index

### Pairwise comparison of diagnostic consistency and dominance between every two scores

The pairwise comparison of diagnostic consistency between every two scores showed that, excellent consistency existed in the pairs of Wells vs Geneva(Kappa 0.753), YEARS vs Padua(Kappa 0.816), YEARS vs IMPROVE(Kappa 0.771), and Padua vs IMPROVE(Kappa 0.789), whereas it did not appear in the other couples. Likewise, the pairwise comparison of diagnostic dominance between every two scores suggested that, there were dominance differences between the two scores of each rest pair except for the aforementioned ones. The pairwise comparison of diagnostic consistency and dominance between every two scores are presented in Table 5.

**Table 5:** Pairwise Comparison of Diagnostic Consistency and Dominance Between Every two Scores

Comparison of scores	Kappa coefficient	*P* value by McNemar’s test
Wells vs Geneva	0.753	0.565
Wells vs YEARS	0.214	0.001
Wells vs PERC	0.185	0.001
Wells vs Padua	0.366	0.001
Wells vs IMPROVE	0.391	0.005
Geneva vs YEARS	0.137	< 0.001
Geneva vs PERC	0.095	< 0.001
Geneva vs Padua	0.286	< 0.001
Geneva vs IMPROVE	0.377	0.004
YEARS vs PERC	0.335	0.016
YEARS vs Padua	0.816	0.873
YEARS vs IMPROVE	0.771	0.645
PERC vs Padua	0.283	0.001
PERC vs IMPROVE	0.211	< 0.001
Padua vs IMPROVE	0.789	0.812

*vs* Versus

### ROC analyses of predictive power for VTE diagnosis of all scores without fixed cutoffs

**Fig. 2:**
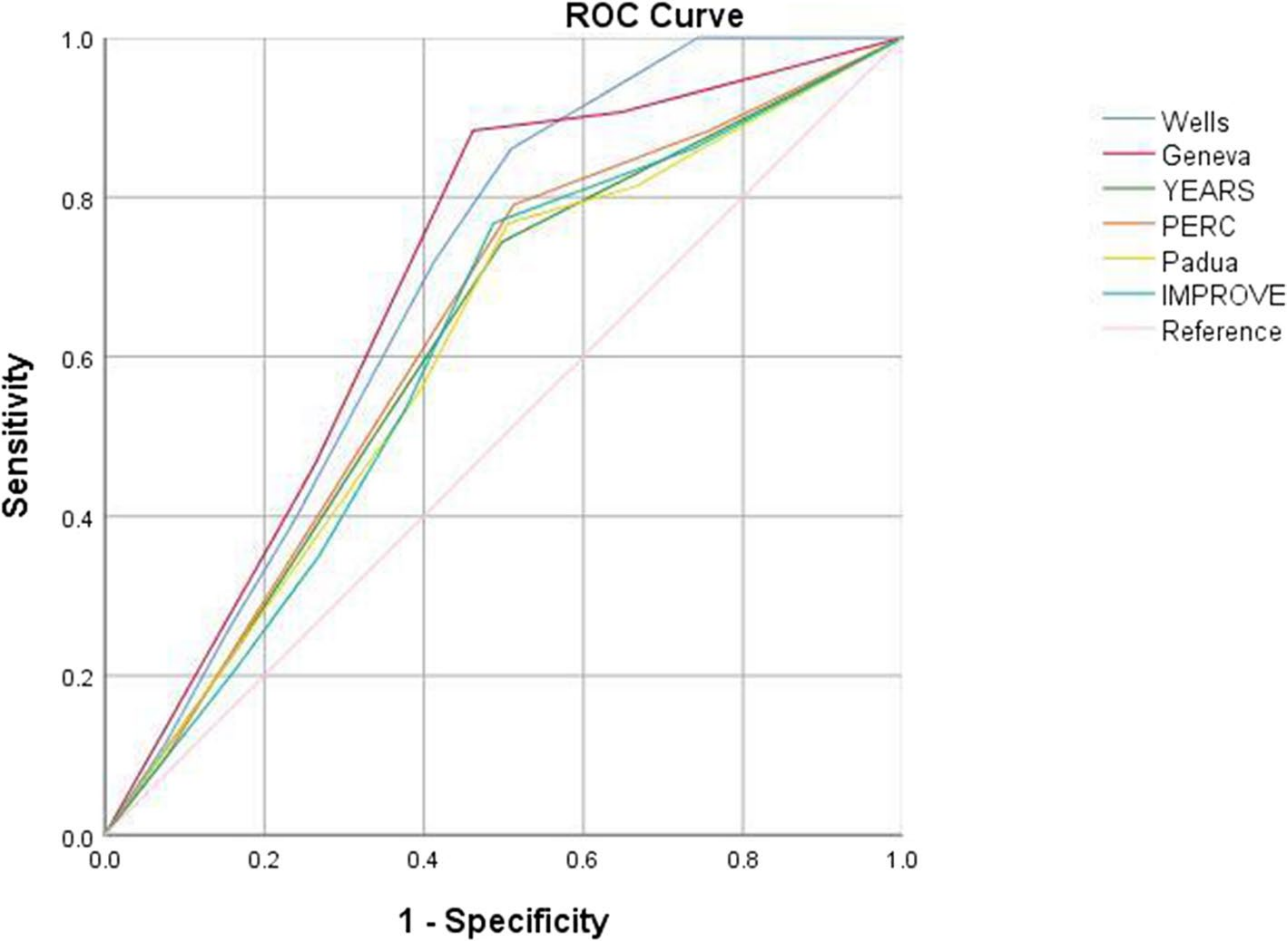
ROC Analyses of Predictive Power for VTE Diagnosis of all Scores Without Fixed Cutoffs. Note: ROC: Receiver operator characteristic, VTE: Venous thromboembolism

By using ROC curve analyses, the predictive power for VTE diagnosis of all scores were reanalyzed without applying the original fixed cutoffs of each RAM in the present study population. The results turned out to indicate that the original cutoffs, sensitivity, specificity, and YI still remained same as those in Table 4 for the Wells, Geneva, and YEARS scores, whereas they changed for the rest scores. With a revised cutoff of 5 or less denoting the VTE-likely, the diagnostic sensitivity, specificity, and YI of PERC were 79.1%, 51.3%, and 0.304, respectively(area under the curve[AUC] 0.631[0.547–0.714], *P* = 0.006). With a revised cutoff of 6 or more denoting the VTE-likely, the diagnostic sensitivity, specificity, and YI of Padua were 76.7%, 50.5%, and 0.272, respectively(AUC 0.613[0.527–0.700], *P* = 0.017). With a revised cutoff of 4 or more denoting the VTE-likely, the diagnostic sensitivity, specificity, and YI of IMPROVE were 76.7%, 49.7%, and 0.264, respectively(AUC 0.614[0.530–0.698], *P* = 0.016). ROC curve analyses of predictive power for VTE diagnosis of all scores without original fixed cutoffs are illustrated in Fig. 2.

## Discussion

The results of the present study revealed that the sequence in the descending order of YI and C-index for the predictive power of VTE diagnosis were the Geneva, Wells, IMPROVE, Padua, YEARS, and PERC scores. No statistical difference with respect to predictive power for VTE diagnosis was found in the pairs of Wells vs Geneva, YEARS vs Padua, YEARS vs IMPROVE, and Padua vs IMPROVE, whereas it presented in the other pairs. In other words, the Geneva and Wells performed best, the PERC performed worst, whereas the others performed intermediately. Revised cutoffs improved the predictive power for VTE diagnosis in the PERC, Padua, and IMPROVE scores. Of note, the absolute predictive performance of all these isolated scores were poor.

The prevalence of VTE in the current cohort was 13.7%, that is basically consistent with previous studies, in which overall VTE event rates in hospitalized medical patients ranged from 10 to 15% [26]. Accordingly, the degree of VTE risk in this study population is representative of nonsurgical hospitalized patients. Most of the items in all these scores were correlated with VTE occurrence in the current patient population, once again validating their eligibility in those scores. The comparison among more than two kinds of these six scores have been rare to date yet. No identical previous studies are available for reference except for some studies analogical to the present one. A recent systematic review compared the capacity of ruling out PE among the Wells, Geneva, YEARS, and PERC scores across different healthcare settings. In the hospitalized healthcare setting, the Wells plus PTP-adjusted D-dimer(sensitivity 95.64%, specificity 39.50%), the Geneva plus PTP-adjusted D-dimer(sensitivity 95.73%, specificity 37.29%), and the YEARS plus PTP-adjusted D-dimer(sensitivity 96.94%, specificity 35.83%) yielded similar diagnostic accuracy [27]. It was basically consistent with the present results, except that the YEARS was inferior to the other two in the current study. Since the aforementioned systematic review incorporated PTP-adjusted D-dimer especially for the YEARS, plus it only targeted PE, it is not appropriate to be regarded as an eligible reference.

Among these six scores, comparison of Wells versus Geneva, and Padua versus IMPROVE were performed most frequently. The results of the comparison between Wells and Geneva were mixed among the related studies. Among them, the results of some previous studies supported the perspective that the Wells and Geneva score had similar prediction accuracy for patients with suspected PE [28–32], whereas the results of some other studies were in favor of that the Wells score was more accurate than the Geneva score [33–38]. In the present study, the predictive power for VTE diagnosis was alike between the Wells and Geneva, albeit the Geneva score seemed slightly better than the Wells score without statistical difference. With respect to Padua versus IMPROVE, several previous studies involving the comparison of them suggested that the predictive power for VTE diagnosis were equally matched between the two scores [39–41]. The results of the present study were consistent with those of previous studies, albeit the IMPROVE seemed slightly better than Padua without statistical significance.

The correlation between predisposing factors or typical indications of VTE in VTE risk scores and VTE occurrence affect their predictive power for VTE diagnosis. The stronger the correlation, the better the predictive power for VTE diagnosis. According to the Table 1, it can be observed that the sum of presence frequency of VTE risk elements in descending order are 30, 29, 28, 26, 26, and 11 times for the Geneva, PERC, Wells, Padua, IMPROVE, and YEARS, respectively. The presence frequency per element in descending order are 4.29, 4.00, 3.71, 3.67, 3.63, and 2.60 for the Geneva, Wells, IMPROVE, YEARS, PERC, and Padua, respectively. The sum of presence frequency of VTE risk elements and the presence frequency per element in authoritative VTE scores especially the latter can embody the relevancy and acceptance degree of these risk elements in VTE risk assessment. According to the Fig. 1, the VTE risk elements which present for at least three times or more are recent immobilization, trauma or surgery(5 times), previous VTE history(5 times), DVT symptoms and/or signs(5 times), hymoptysis(4 times), active cancer(4 times), age (4 times), and heart rate or pulse(3 times).

The determination of cutoffs for risk classification in VTE scores also has an impact on their predictive power for VTE diagnosis. For most VTE scores, the higher the cutoffs, the higher the specificity, the lower the sensitivity, and vice versa. The more appropriate the cutoffs, the better the predictive power for VTE diagnosis. A balance point needs to be quested between missed diagnoses and excessive examinations. Of note, different patient populations with different clinical VTE probability may require different cutoffs. The ratio of VTE-likely cutoffs to total points in descending order are 0.88, 0.33, 0.33, 0.29, 0.20, and 0.17 for the PERC, Geneva, YEARS, Wells, Padua, and IMPROVE RAMs, respectively. Since the PERC score is distinctive among all six scores by reason of that all the items it contains are negative risk factors for VTE occurrence whereas the other five scores all have positive ones for VTE occurrence, its ratio of VTE-likely cutoffs to total points should have been 0.12 which is actually the least in all six scores instead of 0.88, if its items had been set up to be positive risk factors for VTE occurrence.

Ever since the Wells and Geneva score emerged, their role in the PTP prediction of PE have been externally validated in a series of previous studies [2, 6, 9, 27, 37]. The Geneva and Wells have the most(30 times) and third most(28 times) presence frequency of VTE risk elements, as well as highest(4.29) and second highest(4.00) presence frequency per element among all these six scores, respectively. The Geneva and Wells scores both contain the elements of recent immobilization, trauma or surgery(5 times), previous VTE history(5 times), DVT symptoms and/or signs(5 times), hymoptysis(4 times), active cancer(4 times), and heart rate or pulse(3 times), except the Wells score has the element of alternative diagnosis less likely than PE(2 times), whereas the revised Geneva has that of age (4 times). In other words, the Geneva and Wells score especially the former have the most highly-acknowledged risk elements for VTE diagnosis among all six scores. The universally-accepted VTE risk factors in scores which represent most highly-correlated predictors of VTE occurrence could conduce to improve their predictive accuracy for VTE diagnosis. Meanwhile, it can be found that the Wells and Geneva scores are highly similar with each other in composition, of which six elements(26 times) of the total seven ones are identical with each other. This may be accountable for their similar predictive performance in VTE diagnosis. In addition, ROC analyses justified the rationality of their cutoffs. Notwithstanding all this, howsoever, caveat is necessary that the Wells score incorporates a subjective criterion “alternative diagnosis less likely than PE” which is dependent on the experience of clinicians, and is intractable to be standardly operated or imparted, being different from the Geneva.

The Padua and IMPROVE scores are two authoritative ones acknowledged by leading guidelines for medical patients, and have been sufficiently validated in previous external studies [17, 18, 21]. A closer observation at the composition of Padua and IMPROVE revealed that they have the same(26 times) presence frequency of VTE risk elements, whereas the presence frequency per element of the IMPROVE (3.71) is higher than that of the Padua(2.60). These two scores both contain the elements of previous VTE history(5 times), recent immobilization, trauma or surgery(5 times), age(4 times), active cancer(4 times), and thrombophilia(2 times). Their discrepancy in composition is that the Padua score incorporates the elements of ongoing hormonal therapy(2 times), acute infection and/or rheumatologic disorder(1 time), acute myocardial infarction and/or ischemic stroke(1 time), body mass index(1time), and heart and/or respiratory failure(1 time), whereas the IMPROVE incorporates elements of DVT symptoms and signs(5 times) and ICU/CCU stay(1 time). Taken together, the majority of elements(20 of the total 26 times) which are highly-acknowledged risk factors of VTE occurrence are identical between the Padua and IMPROVE. Their similar performance may be attributable to such structural similarity, albeit the IMPROVE seemed slightly better than the Padua without statistical significance.

Overall, the Geneva and Wells generally outperformed the IMPROVE and Padua with respect to the predictive power for VTE diagnosis. These four scores merely share three VTE risk elements which are previous VTE history(5 times), recent immobilization, trauma or surgery(5 times), and active cancer(4 times), whereas had a large proportion of elements not in common. By comparison, the Geneva and Wells both have modifiable risk factors of VTE occurrence like hemoptysis and heart rate or pulse that can reflect the point-of-care status quo of patients, whereas the IMPROVE and Padua do not incorporate these elements. Lack of such elements may abate their predictive power for VTE diagnosis. Of note, notwithstanding these four RAMs all reflect VTE risk, the IMPROVE and Padua were endorsed by the guidelines in terms of VTE prevention or thromboprophylaxis [17, 18], whereas the Geneva and Wells were endorsed in the guidelines of diagnosis and management of PE [2, 9, 10, 15]. The results of present study justified that the IMPROVE and Padua were inferior to the Geneva and Wells with respect to predictive power for VTE diagnosis. Nonetheless, revised cutoffs could improve their performance in certain degree.

The YEARS score is a condensed derivative of the Wells score. Generally, the YEARS algorithm denotes the application of YEARS score in association with a D-dimer level instead of the isolated score alone [2, 24]. Of note, the YEARS in the current study was an isolated score rather than an algorithm since the current study was intended to compare the isolated VTE risk scores without D-dimer. As such, the current results are not applicable to the YEARS algorithm. The YEARS score has only three elements which are DVT symptoms and/or signs(5 times), hemoptysis(4 times) and alternative diagnosis less likely than PE(2 times). Its presence frequency per element is 3.67 which is merely less than those of the Geneva and Wells despite its presence frequency sum of VTE risk elements is only 11. In a retrospective study which compared the predictive accuracy for PE occurrence between the YEARS algorithm(RAM + D-dimer) and the Wells score, the YEARS algorithm was more sensitive than the Wells score (97.44% vs 74.36%), whereas was less specific than the latter(13.97% vs 33.94%). Besides, the YEARS algorithm yielded better negative predictive value than the Wells score (98.0%vs 92.4%). Nevertheless, it was the YEARS algorithm that was employed instead of the isolated YEARS score alone in the study [42]. Accordingly, the study is not an ideal parallel to the current one. In the present study, the diagnostic performance of the isolated YEARS was outperformed by that of the Geneva and Wells, probably due to its excessively simplistic structure, albeit being similar to that of the IMPROVE and Padua. Nevertheless, its cutoff was justified to be appropriate. Of note, the YEARS also has the subjective element which is the “alternative diagnosis less likely than PE”.

The PERC score was originally developed for the PE exclusion among patients with a low clinical probability of PE and has been validated in a randomized controlled trial [43]. It has high sensitivity but low specificity for PE occurrence among patients with intermediate or high clinical probability of PE [2, 44]. Likewise, its predictive power for VTE diagnosis was the worst among all these six scores in the present study in which the subjects were hospitalized patients who carried considerable probability of VTE occurrence, albeit its NPV, FNR, NLR, and DOR were satisfactory yet. Among all these scores, although the presence frequency sum of VTE risk elements in the PERC is 29 times which is only less than that in the Geneva(30 times), whereas its presence frequency per element is 3.63 which is the second least one of among all scores. More importantly, the original cutoff of the PERC that resulted from the patient population with a low clinical probability of PE resulted in its poor predictive power in the current patient population. With the original cutoff of the PERC, substantial excessive unnecessary imaging examinations yielded despite missed diagnoses were drastically avoided, whereas a revised cutoff could improve its performance.

Several limitations need to be acknowledged for the current study. First of all, prospective studies are warranted since the current one was a retrospective review. Secondly, since the current subjects were nonsurgical hospitalized patients, the results may not be applicable to surgical ones, and/or ambulatory outpatients. Besides, generally all nonsurgical hospitalized patients should be included in the evaluation by clinical VTE risk scores. However, only nonsurgical hospitalized patients with suspected VTE were included in this study. Therefore, the results may not be applicable to general nonsurgical hospitalized patients. Thirdly, the Wells and Geneva scores adopted for the present study were simplified version instead of original version, the results might have been different if their original versions had been employed. Likewise, the Wells DVT RAM [6, 16] was not incorporated in the current study either. Last but not least, D-dimer was not involved since the intention of the current study was to compare VTE risk scores. It is worth noting that the absolute performance of each isolated score per se was unsatisfactory(C-index < 0.7 for all), being basically consistent with the results of previous studies [45]. Accordingly, a combination of risk scores and D-dimer is highly recommended by guidelines at present [2]. The results might have been different if D-dimer had been involved.

In conclusion, the comparison of predictive power for VTE diagnosis among six VTE risk scores in guidelines indicates that the Geneva and Wells scores perform best, the PERC score performs worst, whereas the others perform intermediately, in nonsurgical hospitalized patients with suspected VTE. Little difference presents between the Geneva and Wells scores, as well as among the IMPROVE, Padua, and YEARS scores. Revised cutoffs improve the performance of the PERC, Padua, and IMPROVE scores. Nevertheless, the absolute performance of all isolated scores are mediocre. The results may assist clinicians with the selection of relevant scores in the corresponding clinical settings.

## Data Availability

The datasets used and/or analysed during the current study are available from the corresponding authors on reasonable request.

## References

[1] Streiff MB, Holmstrom B, Angelini D, et al. Cancer-Associated Venous Thromboembolic Disease, Version 2.2021, NCCN Clinical Practice Guidelines in Oncology. J Natl Compr Canc Netw. 2021;19(10):1181–1201. 10.6004/jnccn.2021.0047 34666313

[2] Konstantinides SV, Meyer G, Becattini C (2020). 2019 ESC Guidelines for the diagnosis and management of acute pulmonary embolism developed in collaboration with the European Respiratory Society (ERS). Eur Heart J.

[3] Raskob GE, Angchaisuksiri P, Blanco AN (2014). Thrombosis: a major contributor to global disease burden. Arterioscler Thromb Vasc Biol.

[4] Wendelboe AM, Raskob GE. Global burden of thrombosis: epidemiologic aspects.Circ Res 2016;118:1340–1347. 10.1161/CIRCRESAHA.115.306841 27126645

[5] Keller K, Hobohm L, Ebner M (2020). Trends in thrombolytic treatment and outcomes of acute pulmonary embolism in Germany. Eur Heart J.

[6] Khan F, Tritschler T, Kahn SR, Rodger MA (2021). Venous thromboembolism. Lancet.

[7] Penaloza A, Verschuren F, Meyer G (2013). Comparison of the unstructured clinician gestalt, the wells score, and the revised Geneva score to estimate pretest probability for suspected pulmonary embolism. Ann Emerg Med.

[8] Sanders S, Doust J, Glasziou P (2015). A systematic review of studies comparing diagnostic clinical prediction rules with clinical judgment. PLoS ONE.

[9] Lim W, Le Gal G, Bates SM (2018). American Society of Hematology 2018 guidelines for management of venous thromboembolism: diagnosis of venous thromboembolism. Blood Adv.

[10] Torbicki A, Perrier A, Konstantinides S (2008). Guidelines on the diagnosis and management of acute pulmonary embolism: the Task Force for the Diagnosis and Management of Acute Pulmonary Embolism of the European Society of Cardiology (ESC). Eur Heart J.

[11] Wells PS, Anderson DR, Rodger M (2000). Derivation of a simple clinical model to categorize patients probability of pulmonary embolism: increasing the models utility with the SimpliRED D-dimer. Thromb Haemost.

[12] Gibson NS, Sohne M, Kruip MJ (2008). Further validation and simplification of the Wells clinical decision rule in pulmonary embolism. Thromb Haemost.

[13] Le Gal G, Righini M, Roy PM (2006). Prediction of pulmonary embolism in the emergency department: the revised Geneva score. Ann Intern Med.

[14] Klok FA, Mos IC, Nijkeuter M (2008). Simplification of the revised Geneva score for assessing clinical probability of pulmonary embolism. Arch Intern Med.

[15] Kearon C, Akl EA, Comerota AJ (2012). Antithrombotic therapy for VTE disease: Antithrombotic Therapy and Prevention of Thrombosis, 9th ed: American College of Chest Physicians Evidence-Based Clinical Practice Guidelines. Chest.

[16] Bates SM, Jaeschke R, Stevens SM (2012). Diagnosis of DVT: Antithrombotic Therapy and Prevention of Thrombosis, 9th ed: American College of Chest Physicians Evidence-Based Clinical Practice Guidelines. Chest.

[17] Kahn SR, Lim W, Dunn AS (2012). Prevention of VTE in nonsurgical patients: Antithrombotic Therapy and Prevention of Thrombosis, 9th ed: American College of Chest Physicians Evidence-Based Clinical Practice Guidelines. Chest.

[18] Schünemann HJ, Cushman M, Burnett AE (2018). American Society of Hematology 2018 guidelines for management of venous thromboembolism: prophylaxis for hospitalized and nonhospitalized medical patients. Blood Adv.

[19] Gould MK, Garcia DA, Wren SM (2012). Prevention of VTE in nonorthopedic surgical patients: Antithrombotic Therapy and Prevention of Thrombosis, 9th ed: American College of Chest Physicians Evidence-Based Clinical Practice Guidelines. Chest.

[20] Nendaz M, Spirk D, Kucher N, et al. Multicentre validation of the Geneva Risk Score for hospitalised medical patients at risk of venous thromboembolism. Explicit ASsessment of Thromboembolic RIsk and Prophylaxis for Medical PATients in SwitzErland (ESTIMATE). Thromb Haemost. 2014;111(3):531–538. 10.1160/TH13-05-0427 24226257

[21] Darzi AJ, Repp AB, Spencer FA (2020). Risk-assessment models for VTE and bleeding in hospitalized medical patients: an overview of systematic reviews. Blood Adv.

[22] Gibson NS, Sohne M, Kruip MJ (2008). Further validation and simplification of the Wells clinical decision rule in pulmonary embolism. Thromb Haemost.

[23] Klok FA, Mos IC, Nijkeuter M (2008). Simplification of the revised Geneva score for assessing clinical probability of pulmonary embolism. Arch Intern Med.

[24] van der Hulle T, Cheung WY, Kooij S (2017). Simplified diagnostic management of suspected pulmonary embolism (the YEARS study): a prospective, multicentre, cohort study. Lancet.

[25] Freund Y, Cachanado M, Aubry A (2018). Effect of the Pulmonary Embolism Rule-Out Criteria on Subsequent Thromboembolic Events Among Low-Risk Emergency Department Patients: The PROPER Randomized Clinical Trial. JAMA.

[26] Spyropoulos AC (2010). Risk assessment of venous thromboembolism in hospitalized medical patients. Curr Opin Pulm Med.

[27] Geersing GJ, Takada T, Klok FA (2022). Ruling out pulmonary embolism across different healthcare settings: A systematic review and individual patient data meta-analysis. PLoS Med.

[28] Chagnon I, Bounameaux H, Aujesky D (2002). Comparison of two clinical prediction rules and implicit assessment among patients with suspected pulmonary embolism. Am J Med.

[29] Klok FA, Kruisman E, Spaan J (2008). Comparison of the revised Geneva score with the Wells rule for assessing clinical probability of pulmonary embolism. J Thromb Haemost.

[30] Wong DD, Ramaseshan G, Mendelson RM (2011). Comparison of the Wells and Revised Geneva Scores for the diagnosis of pulmonary embolism: an Australian experience. Intern Med J.

[31] Esiéné A, Tochie JN, Metogo JAM, Etoundi PO, Minkande JZ (2019). A comparative analysis of the diagnostic performances of four clinical probability models for acute pulmonary embolism in a sub-Saharan African population: a cross-sectional study. BMC Pulm Med.

[32] Coelho J, Divernet-Queriaud M, Roy PM, Penaloza A, Le Gal G, Trinh-Duc A (2020). Comparison of the Wells score and the revised Geneva score as a tool to predict pulmonary embolism in outpatients over age 65. Thromb Res.

[33] Calisir C, Yavas US, Ozkan IR (2009). Performance of the Wells and Revised Geneva scores for predicting pulmonary embolism. Eur J Emerg Med.

[34] Penaloza A, Melot C, Motte S (2011). Comparison of the Wells score with the simplified revised Geneva score for assessing pretest probability of pulmonary embolism. Thromb Res.

[35] Di Marca S, Cilia C, Campagna A (2015). Comparison of Wells and Revised Geneva Rule to Assess Pretest Probability of Pulmonary Embolism in High-Risk Hospitalized Elderly Adults. J Am Geriatr Soc.

[36] Guo DJ, Zhao C, Zou YD, Huang XH, Hu JM, Guo L (2015). Values of the Wells and revised Geneva scores combined with D-dimer in diagnosing elderly pulmonary embolism patients. Chin Med J (Engl).

[37] Hendriksen JM, Geersing GJ, Lucassen WA (2015). Diagnostic prediction models for suspected pulmonary embolism: systematic review and independent external validation in primary care. BMJ.

[38] Shen JH, Chen HL, Chen JR, Xing JL, Gu P, Zhu BF (2016). Comparison of the Wells score with the revised Geneva score for assessing suspected pulmonary embolism: a systematic review and meta-analysis. J Thromb Thrombolysis.

[39] Greene MT, Spyropoulos AC, Chopra V (2016). Validation of Risk Assessment Models of Venous Thromboembolism in Hospitalized Medical Patients. Am J Med.

[40] Blondon M, Spirk D, Kucher N (2018). Comparative Performance of Clinical Risk Assessment Models for Hospital-Acquired Venous Thromboembolism in Medical Patients. Thromb Haemost.

[41] Moumneh T, Riou J, Douillet D (2020). Validation of risk assessment models predicting venous thromboembolism in acutely ill medical inpatients: A cohort study. J Thromb Haemost.

[42] Abdelaal Ahmed Mahmoud M Alkhatip A, Donnelly M, Snyman L, et al. YEARS Algorithm Versus Wells' Score: Predictive Accuracies in Pulmonary Embolism Based on the Gold Standard CT Pulmonary Angiography. Crit Care Med. 2020;48(5):704–708. 10.1097/CCM.0000000000004271 32079894

[43] Penaloza A, Soulié C, Moumneh T (2017). Pulmonary embolism rule-out criteria (PERC) rule in European patients with low implicit clinical probability (PERCEPIC): a multicentre, prospective, observational study. Lancet Haematol.

[44] Freund Y, Cachanado M, Aubry A (2018). Effect of the Pulmonary Embolism Rule-Out Criteria on Subsequent Thromboembolic Events Among Low-Risk Emergency Department Patients: The PROPER Randomized Clinical Trial. JAMA.

[45] Pandor A, Tonkins M, Goodacre S (2021). Risk assessment models for venous thromboembolism in hospitalised adult patients: a systematic review. BMJ Open.

